# First Ever Whole Genome Sequencing and De Novo Assembly of the Freshwater Angelfish, *Pterophyllum scalare*

**DOI:** 10.17912/micropub.biology.000654

**Published:** 2022-10-18

**Authors:** Indeever Madireddy

**Affiliations:** 1 BioCurious, Santa Clara, CA, USA; 2 BASIS Independent Silicon Valley, San Jose, CA, USA

## Abstract

This research work is the first to ever sequence and perform a de novo assembly of the genome of the freshwater angelfish, *Pterophyllum scalare* . The final genome assembly consisted of 15,486 contigs and was 734.79 Mb in size with an 86.5% BUSCO score. Functional annotation of the genome revealed 24,247 protein-coding sequences related to other fish species. 14,329 (59%) of the identified genes were orthologous to *Archocentrus centrarchus* , a closely related South American cichlid.

**Figure 1. Long-read nanopore sequencing of angelfish DNA leads to a robust genome assembly:**
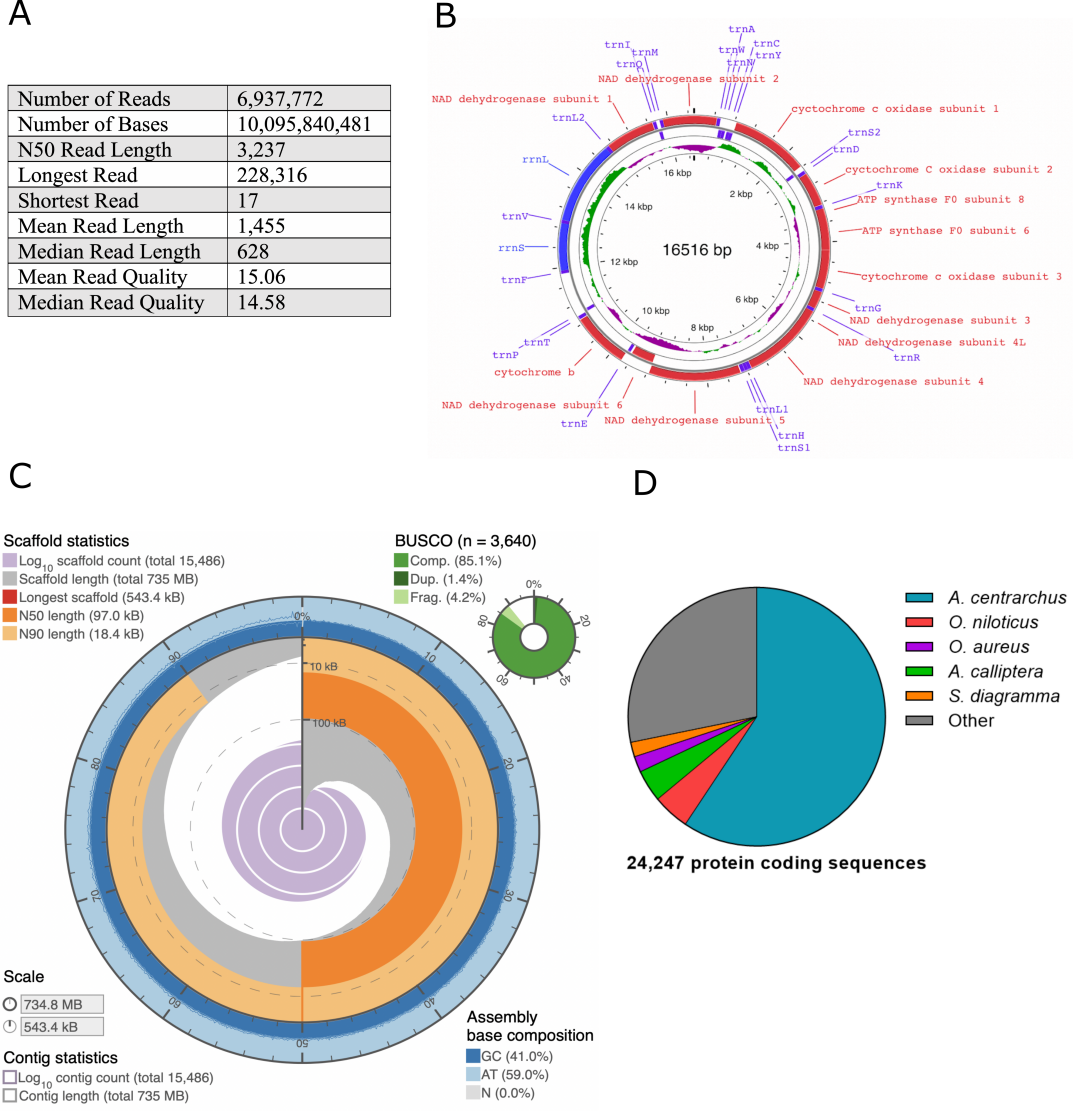
**A)** Table depicting the read statistics of the nanopore sequencing including the number of collected reads, the mean read length, and mean read quality. **B)** Angelfish mitochondrial genome annotated with the 37 genes. Violet text refers to tRNAs, red text refers to protein-coding genes, and blue text corresponds to rRNAs. The green histogram indicates + GC skew while the purple histogram indicates -GC skew. **C)** Snail plot showing the assembled nuclear genome with its corresponding statistics. **D)** Orthologous genes were most commonly identified in the following organisms: *Archocentrus centrarchus* , *Oreochromis niloticus, Oreochromis aureus, Astatotilapia calliptera* , and *Simochromis diagramma* .

## Description

The freshwater angelfish, *Pterophyllum scalare,* is a popular freshwater cichlid kept by aquarium hobbyists around the world. Originally from South America, these fish are well known for their monogamous breeding patterns and thorough parental care of offspring (Cacho et al., 2007). Although the behaviors of the angelfish have been well studied, very little is known about the nuclear genetics of the angelfish as its genome has never been fully sequenced and assembled (Gómez-Laplaza and Gerlai, 2020). Cichlids are of especial importance to biomedical research, for they have been used as model organisms to study craniofacial variation and neurobiology (Powder and Albertson, 2016; Maruska and Fernald, 2018). Investigating the genome of the angelfish may enable its use as a model organism for further biological research. In this work, I sequenced, assembled, and annotated the complete genome of the freshwater angelfish in addition to the full mitochondrial genome with Oxford Nanopore Technologies (Lu et al., 2016).

With the MinION MK1B device, 6.94 million sequencing reads (figure A) and an estimated 10.1 gigabases at a 3.24 kb N50 read length were collected (Steinig and Coin, 2022). Two flow cells (R10.4) were used to collect this sequencing data, and the flow cells were run for 72 hours each. The reads collected had a mean read quality of 15.06 and a median read quality of 14.58 corresponding to an estimated 97% sequencing accuracy. Reads were collected at an average translocation speed of 220 bases per second.

Collected reads were then screened to identify potential contaminant organisms in the sequencing data. The kraken2 tool (ver. 2.1.2) identified that *Pseudomonas aeruginosa,* a common opportunistic aquatic pathogen, was the largest contaminant of the sequencing reads (Wood and Salzberg, 2014; Souza et al., 2019).

The mitochondrial genome (figure B) of the angelfish was assembled from the sequencing reads. All 37 conserved mitochondrial genes including 2 rRNAs, 13 genes and 22 tRNAs common to eukaryotic organisms were identified, indicating a complete and robust assembly. This new assembly was found to be 25 bp longer than the reference mitochondrial assembly, with a 99.1% similarity.

The final nuclear genome assembly (figure C) consisted of 15,486 contigs totaling 734.79 Mb with a final BUSCO score of 86.5% and a 41% GC content (Simão et al., 2019). The genome size and GC content is similar to other fish species such as the Asian seabass ( *Lates calcarifer* ) and the Nile tilapia ( *Oreochromis niloticus* ) (Lu and Luo, 2020). The N50 contig length of the assembled genome was 96,962 bp, and the longest contig was 543,394 bp. Repeatmasker (ver. 4.1.1) masked 12.47% of the genome containing simple repeat sequences (Chen, 2004). An interactive version of the whole genome can be found here: https://indeeverm.github.io/assembly-stats/

NCBI blastp (ver. 2.12.0) performed functional annotation of the genome through the GenSAS platform (States and Gish, 1994; Humann et al, 2019). 24,247 unique protein-coding sequences orthologous to other species were identified in the angelfish genome against the refseq vertebrate-other database. A majority of genes, 59%, were orthologous to *Archocentrus centrarchus,* a closely related South American cichlid (figure D). Timetree suggests that *A. centrarchus* and *P. scalare* diverged between 28.7 to 72.4 million years ago (Kumar et al., 2017).

Future work would involve RNA sequencing of the angelfish to build an appropriate transcriptome of the organism. Illumina sequencing could also be performed to improve the current assembly.

## Methods

*Angelfish*

The angelfish used in this work died of natural causes prior to the start of experimentation and was neither euthanized nor harmed in any way for the purpose of this research. Although IACUC approval was not required, all US regulations were followed in the collection and handling of the biological material. Angelfish tissue was obtained from an angelfish raised by the author from birth. Angelfish muscle and skin tissue were collected post-mortem with a punch biopsy. Fish tissue was stored in a DNA shield at -80°C until use.

*Angelfish Genomic DNA Extraction*

Angelfish genomic DNA was extracted with the NEB Monarch genomic DNA purification kit. The standard protocol for tissue samples was followed with the following specifications. 30 mg of angelfish muscle tissue was lysed with the provided tissue lysis buffer and proteinase K for 1.5 hours at 56°C and shaking at 2000 rpm. Samples were vortexed every 5 minutes for 5 seconds during lysis. Cell debris was pelleted at 12000 x g after lysis and the supernatant was transferred to a new microcentrifuge tube. RNAse A addition was not skipped to minimize RNA carryover. Genomic DNA was washed three times with wash buffer 1 and eluted in 60μL of TE buffer heated to 70°C. DNA concentration was measured with a Denovix DS-11 spectrophotometer.

Extracted genomic DNA was then run on a 1 percent TBE agarose gel in pulsed-field gel electrophoresis in order to verify DNA length and quality. Samples were run in the CHEF-DR II system for 20 hours at 3.5 V/cm with a pulse shift every 25 seconds. Genomic DNA fragments were approximately 20-40kb in length.

*Library prep*

The standard ligation library prep (SQK-LSK112) was performed on the angelfish genomic DNA following the manufacturer’s protocol.

*Base-calling*

Base-calling was performed at high accuracy by the Guppy base-caller (ver. 6.1.5).

*Nuclear Genome Assembly*

I performed whole-genome assembly with the flye de novo assembler (ver. 2.9) built into the Project Galaxy bioinformatics tool (ver. 22.01) with one round of polishing (Kolmogorov et al., 2019; Afgan et al., 2019) . Shorter reads (< 1000 bp) were eliminated as they did not improve the assembly and increased computation time. Only reads with a quality score above 9 were used in the assembly. A BUSCO (Benchmarking Universal Single-Copy Orthologs) score, a basis for the completeness of a genome, was calculated for the assembly with the reference being the *Actinopterygii* lineage and was found to be 88% for the initial Flye assembly. Neither polishing the genome with Racon nor including lower quality reads in the assembly improved this score (Vaser et al., 2017). Smaller-sized contigs (< 5000 bp) were then removed from the final assembly as they ware found to not contribute to the BUSCO score. Kraken2 was used again to identify and remove contaminating contigs. Mitochondrial contigs were also removed from the nuclear genome assembly. The genome snail plot was generated using https://github.com/rjchallis/assembly-stats (ver. 17.02).

*Mitochondrial Genome Assembly*

I assembled the mitochondrial genome of the angelfish against one already assembled by Hao et al (Hao et al., 2016). The Epi2Me fastq custom alignment program (ver. 3.4.2) was used to align the 6.94 million collected reads against the mitochondrial reference genome to filter out unnecessary genomic reads. The Shasta de novo assembler (ver. 0.10.0) then assembled the remaining mitochondrial reads. This assembly resulted in a 16,516 base pair mitochondrial genome (Shafin et al., 2020). The MITOS web server (http://mitos.bioinf.uni-leipzig.de/index.py) was used to annotate the genome (Bernt et al., 2013). This plot was generated with https://proksee.ca (ver. 1.0.0).

*Additional Information*

A pipeline for this work is available on GitHub: https://github.com/IndeeverM/Angelfish-Genome-Assembly .

This Whole Genome Shotgun project has been deposited at DDBJ/ENA/GenBankunder the accession JAMQGT000000000. The version described in this paper is version JAMQGT020000000.

## Reagents

**Table:** 

Kit	Use
Nanopore Ligation Sequencing Kit (SQK-LSK112)	Library Prep
Flow Cell Wash Kit (EXP-WSH004)	Washing of Flow Cell
NEB Monarch Genomic DNA Purification Kit	Extracting Angelfish Genomic DNA
